# Genetic diversity, QoI fungicide resistance, and mating type distribution of *Cercospora sojina*—Implications for the disease dynamics of frogeye leaf spot on soybean

**DOI:** 10.1371/journal.pone.0177220

**Published:** 2017-05-09

**Authors:** Sandesh Kumar Shrestha, Alicia Cochran, Alemu Mengistu, Kurt Lamour, Arturo Castro-Rocha, Heather Young-Kelly

**Affiliations:** 1 Department of Entomology and Plant Pathology, University of Tennessee, Knoxville, Tennessee, United States of America; 2 Department of Entomology and Plant Pathology, University of Tennessee, Jackson, Tennessee, United States of America; 3 United States Department of Agriculture, Agriculture Research Service, Jackson, Tennessee, United States of America; 4 Departamento de Ciencias Químico-Biológicas, Universidad Autónoma de Cd. Juárez, Chihuahua, Mexico; Georg-August-University of Göttingen Institute of Microbiology & Genetics, GERMANY

## Abstract

Frogeye leaf spot (FLS), caused by *Cercospora sojina*, causes significant damage to soybean in the U.S. One control strategy is the use of quinone outside inhibitor (QoI) fungicides. QoI resistant isolates were first reported in Tennessee (TN) in 2010. To investigate the disease dynamics of *C*. *sojina*, we collected 437 *C*. *sojina* isolates in 2015 from Jackson and Milan, TN and used 40 historical isolates collected from 2006–2009 from TN and ten additional states for comparison. A subset of 186 isolates, including historical isolates, were genotyped for 49 single nucleotide polymorphism (SNP) markers and the QoI resistance locus, revealing 35 unique genotypes. The genotypes clustered into three groups with two groups containing only sensitive isolates and the remaining group containing all resistant isolates and a dominant clonal lineage of 130 isolates. All 477 *C*. *sojina* isolates were genotyped for the QoI locus revealing 344 resistant and 133 sensitive isolates. All isolates collected prior to 2015 were QoI sensitive. Both mating type alleles (*MAT1-1-1* and *MAT1-2*) were found in Jackson and Milan, TN and recovered from single lesions suggesting sexual recombination may play a role in the epidemiology of field populations. Analysis of *C*. *sojina* isolates using SNP markers proved useful to investigate population diversity and to elaborate on diversity as it relates to QoI resistance and mating type.

## Introduction

Frogeye leaf spot (FLS) of soybean (*Glycine max* Merr.), caused by the fungal pathogen *C*. *sojina* Hara, was first identified in Japan in 1915 and South Carolina, United States in 1924 [1, 2]. FLS is an important foliar disease of soybean in the US and symptoms can appear on stems, pods, and seeds [3]. Initial symptoms appear as small, light brown circular spots which develop a darkish brown to reddish margin [4]. As the infected leaf area reaches 50%, the leaves blight, wither and fall prematurely. Conidia, produced on conidiophore are primary and secondary sources of inoculum and are produced on infected leaves, stems, and pods [2]. Warm temperature and frequent rainfall promote severe disease development and fully expanded leaves have smaller lesions compared to younger leaves [2].

According to the food and agriculture organization (FAO), the United States is the world’s leading producer of soybean (second only to corn) and produced 106.9 million metric tons of soybeans in 2015 [5]. Yield losses are due to reduced photosynthetic area and the premature defoliation of leaves [3, 6]. In the US, FLS has been predominately a disease of southern warm and humid regions [3, 7], but has been reported in northern states, such as Iowa in 1999, Wisconsin in 2000 [8] and Ohio in 2006 [9]. Damage depends on cultivar and location, with yield losses ranging from 10% to more than 60% [4, 10–12].

FLS is a polycyclic disease and remains active throughout the growing season [11, 13]. Spores are dispersed by the wind and water splashes [11]. *C*. *sojina* can overwinter in plant debris and may survive for two years in northern environments [9, 14]. Greenhouse trials suggest *C*. *sojina* may survive on alternate hosts in the absence of soybean [15]. Control is accomplished through the use of cultural practices, fungicides and planting of resistant cultivars. Cultivars with genetic resistance to FLS can be effective and three resistance genes, *Rcs* (Resistant to *C*. *sojina*), have been identified including *Rcs1* [16], *Rcs2* [17] and *Rcs3* [18]. The *Rcs3* gene confers resistance against race 5 and all known races of *C*. *sojina* present in the U.S. [18, 19]. Crop rotation for two years is thought to reduce infection and limit disease severity [14, 20]. In addition, the use of pathogen-free seeds and application of fungicides are used to decrease disease severity [20]. While these management practices can still be successful, they have placed selection pressures on *C*. *sojina* populations and resulted in isolates that have overcome genetic resistance, namely the *Rcs1* and *Rcs2* genes [16, 17, 21]. Similarly, *C*. *sojina* isolates have developed resistance to the quinone outside inhibitor (QoI) fungicide group [22].

Previous studies characterized population diversity of *C*. *sojina* using AFLP and simple sequence repeat (SSR) markers and there is evidence for sexual outcrossing in field populations [13, 23]. Currently, there are no universally accepted soybean differentials and reports of race diversity include 12 races of *C*. *sojina* in the US, 22 races in Brazil and 14 races in China [20]. Use of the same 12 soybean differentials produced differing numbers of proposed races in two separate studies [3, 9].

Our primary objective was to investigate the dynamics of QoI resistance on eight cultivars of soybean that were fungicide treated and untreated at two locations in Tennessee in 2015. Supporting objectives included the development of novel SNP markers to characterize genotypic diversity, comparison of the 2015 isolates to isolates collected previously and from surrounding states, and an assessment of mating type loci at Tennessee locations and within individual lesions.

## Materials and methods

### Sample collection, single-lesion isolation, and DNA extraction

This study was carried out on private land and the owner gave permission to collect all samples. In 2015, soybean leaves with typical FLS lesions were collected from research plots at two locations in Tennessee (Milan and Jackson). In total, 437 isolates, 203 from Jackson and 234 from Milan, were collected from eight fungicide treated and non-treated Maturity group III soybean cultivars (Table 1). Cultivars were planted in four rows with 76.2 cm row spacing (30-inch row spacing) in 7.6 m (25 ft) long plots in a randomized complete block design with four replications. Plots were split. Two rows were untreated, and two rows were treated at the R3 growth stage (beginning pod) with Quadris Top SB at 8 fl oz/a (0.12 kg a.i./ha Azoxystrobin and 0.07 kg a.i./ha Difenoconazole, Syngenta Corp., Basel, Switzerland). A single isolate of *C*. *sojina* was obtained from a single lesion from an individual leaflet. Sporulation was induced by incubating leaves in a plastic bag with moist towels at room temperature (approximately 24°C/72°F). Spores were harvested with a flame-sterilized needle using a dissecting microscope and 8–10 spores transferred to RA-V8 agar media (rifampicin 25 ppm, ampicillin 100 ppm, 160 mL unfiltered V8 juice, 3 gm calcium carbonate and 840 mL water). Observations were made daily and contaminated sectors removed. After seven days, single-lesion isolates of *C*. *sojina* were transferred to new plates. In addition, a set of 40 isolates from 10 different states, collected between 2006 and 2009, were included (Table 2).

**Table 1:** Soybean cultivars and number of *Cercospora sojina* isolates recovered from fungicide treated and untreated cultivars in Jackson and Milan, Tennessee.

Cultivar ID	Cultivars	Jackson		Milan		Total
		Treated	Untreated	Treated	Untreated	
C1	VAR Armor 37-R33 RR2	17	11	21	4	53
C2	VAR Asgrow AG3832 GENRR2Y	7	15	20	14	56
C3	VAR Beck's 393R4	0	0	0	3	3
C4	VAR Croplan R2C 3984	19	14	11	14	58
C5	VAR Mycogen 5N393R2 RR2 g	12	20	17	28	77
C6	VAR Terral REV 39A35	10	14	13	16	53
C7	VAR USG 73P93R	22	6	13	21	62
C8	VAR Warren Seed 3780 R2Y It	14	22	13	26	75

**Table 2:** Number of *Cercospora sojina* isolates collected from Jackson and Milan, Tennessee in 2015 and historical isolates from 11 states.

Location	No. of Samples	Year
Jackson, TN	203	2015
Milan, TN	234	2015
Alabama	5	2006
Arkansas	5	2006
Florida	1	2006
Georgia	4	2006
Iowa	1	2006
Illinois	2	2006/2009
Louisiana	1	2006
Mississippi	6	2006
South Carolina	2	2006/2009
Tennessee	12	2007
Wisconsin	1	2006

For DNA extraction, single-leision isolates were grown in 24-deepwell plates (Fisher Scientific) with 1 mL RA-V8 liquid broth (same as above, minus the agar) per well. DNA was extracted as described by Lamour and Finley (2006). Briefly, this includes harvesting mycelium from the broth cultures into a 96-well 2 mL deepwell plate pre-loaded with 3–5 sterilized 3 mm glass beads. The plates are freeze dried and the dried mycelium powdered using a Mixer-Mill bead beating device (Qiagen). The powdered mycelium was then lysed and a standard glass fiber spin-column DNA extraction completed. The resulting genomic DNA was visualized on a 1% gel and quantified using a Qubit device (Thermo Fisher Scientific Inc.) using the high-sensitivity assay.

### SNP marker discovery and Targeted-sequencing based genotyping

Whole genome sequencing was accomplished for three FLS isolates, two from the collection of Dr. Dan Philips at the Univesity of Georgia and one from Tennessee. These include isolate FLS19 (TN10) recovered from the Georgia Experiment Station in 1994, FLS21 (TN85) recovered from Charleston Mississippi in 1994, and FLS11 (CS10117) recovered from Milan, Tennessee in 2010. Genomic DNA was extracted from freeze-dried and powdered mycelium using a standard phenol-chloroform approach and the resulting DNA was submitted to the Beijing Genomics Institute in China for 2x100 paired-end sequencing on an Illumina HiSeq2000 device. *De novo* assembly, read mapping and SNP discovery were accomplished with CLC Genomics workbench 7 (Qiagen). As there was no public reference genome available at the time, FLS21 was *de novo* assembled using the default settings in CLC and the resulting contigs used as a reference genome. All open reading frames (ORFs) longer than 300 amino acids were predicted using CLC and annotated onto the FLS21 contigs. The raw reads from FLS11 and FLS19 were then mapped to the draft reference (separately), and putative single nucleotide variants (SNVs) identified at sites with at least 20X coverage and an alternate allele frequency greater than 90%.

A subset of the SNVs were chosen from the largest contigs for further genotyping using a targeted sequencing approach. Custom Perl scripts (www.github.com/sandeshsth) were used to extract the flanking sequences for the panel of SNPs and primers were designed using BatchPrimer3 v1.0 (http://probes.pw.usda.gov/batchprimer3/) to amplify targets between 80 and 120bp in length. Primers for 50 SNPs, including the mitochondrial QoI resistance locus, are summarized in Table 3. Primer sequences and genomic DNA were sent to Floodlight Genomics (Knoxville, TN) for processing as part of a non-profit Educational and Research Outreach Program (EROP) that provides targeted-sequencing services at cost for academic researchers. Floodlight Genomics uses an optimized Hi-Plex approach to amplify targets in multiplex PCR reactions and then prepares libraries for sequencing on an NGS device [24]. The services include testing of primers to determine optimal multiplex mixtures followed by standard PCR amplification that includes the addition of sample-specific barcode sequences. The resulting amplicons are prepared for sequencing using PCR-free library construction and the samples presented here were sequenced on an Illumina HiSeq3000 device running a 2x150bp configuration per the manufacturer’s directions (Illumina). The resulting sequences were demultiplexed based on the sample-specific barcodes and then mapped to the reference contigs and genotypes assigned for loci with at least 6X coverage and an alternate allele frequency greater than 90%.

**Table 3:** Summary data for 49 nuclear SNP loci and the mitochondrial QoI-resistance locus.

Locus	Contig_SNPposition	Ref	Alt Allele	Forward Primer (5’-3’)	Reverse Primer (5’-3’)
L01	Cs_85_10076	C	T	TTGAGCCTCCCGATGAAC	TCACAAGATCGAACCATCCA
L02	Cs_248_62315	G	A	ATGGCGAGACCGTTCAGT	GGGCCGCGAGTACAATTA
L03	Cs_24_59400	A	G	CGAACCTTGGCTCCTTGA	ACAGGATCGCAGCCAGAC
L04	Cs_30_57551	T	G	TGCGAGTTTGTCCAGGTG	ATATCCCGCGGAATCCAT
L05	Cs_89_38914	A	G	ACCAGCCTCCACATCGAA	AAGCCACAACGTTGCACA
L06	Cs_131_36454	C	A	GATCCAGGATGACCAGCAG	TGCTCCCCATCATGACCA
L07	Cs_304_59625	C	T	CAGCCACTGATGGCACAA	TTGAGCAAACAGCACACACA
L08	Cs_290_54459	C	T	GGCATCCTTCGCTACGTG	AGTCCAAAGAGCGCGAAG
L09	Cs_25_109428	A	G	TGGCTTACGGAACAGACCA	CGTCCGATTGCAGCACTA
L10	Cs_189_55629	G	A	ATCGAGCTTGCGGTTGAC	CGCATCTCGATGCACCT
L11	Cs_70_9315	A	G	GAGGGAATGGGGATGGAT	GAGCGTTTCACTGCCCATA
L12	Cs_181_27548	C	T	GCGATGGCTGTTGAGGTT	CGATCGCATCAGCACTTG
L13	Cs_125_13742	A	G	AATGCGATCCCGGTCAC	TCCTCCACCACCGTCAAC
L14	QoI locus	G (sensitive)	C (resistant)	GGGTTATGTTTTACCTTACGGACAAATG	GTCCTACTCATGGTATTGCACTCA
L15	Cs_269_60141	G	A	CACATTACCGGGGACGAA	CCGGATGCTGCTGGTATC
L16	Cs_386_7516	C	G	GCAATCCGCTCTCAGTCC	CAAGTACAGCCCGCTCCA
L17	Cs_42_10502	A	G	AAGCTTGAGCCCTTTTTGC	TAGGACGGCCAAGCCATA
L18	Cs_42_106606	G	A	GACAACCGCTACGCATCC	GAGGACGACGAGGCAGAA
L19	Cs_290_2709	G	A	GGGTGGCTATCGTGTTGC	TATTCCTGCACGGCTTCG
L20	Cs_419_55098	A	G	CCAGATCGCAAGCCACTC	GCTCGATCCTCGCAATGT
L21	Cs_68_11048	C	T	GCGACACTATGGGATCAAGA	ATGCCAGCGAACTTCCAG
L22	Cs_131_60081	T	G	ACCAAGGTCCTCGACACG	GAGCAAAACCAGCCTTGG
L23	Cs_274_24784	T	C	TCACCACACCTGGCACAC	CAGTCCATCCAAGGTCAGG
L24	Cs_181_12610	C	T	CGGGAACGGAAATCGAG	GCCAGGCCTGTTCTTCG
L25	Cs_1_66202	A	G	CATCGGATCCAGTACCGAGT	AACCGGTCGGACGTCTTT
L26	Cs_128_27517	C	T	CGAGCATCCCAGATCGAG	GCTCGTCTCCCACACCTC
L27	Cs_343_52696	G	A	GGTTGCCGAAATGCAGTG	CCGTAATCGATCCGGCTA
L28	Cs_119_105430	A	T	ACGGCCAAGCTATCATGC	GCGTCCCTCCGGATACAT
L29	Cs_142_22879	G	A	CGAATGAAAGGCCTCGTG	TCATCCCGTCTTCGCAAC
L30	Cs_142_70677	T	C	CCCCAAGAAACCCTCTGG	GAACCAGTGCGCGAGAA
L31	Cs_155_10397	A	G	GGTCGAAGAAGCGCAAGA	ACCGCTCCACAAGCTCCT
L32	Cs_157_19613	G	A	TGTATCGGTGCGCATTTG	GGAGGGGTCAGAGCAGGT
L33	Cs_157_39528	C	T	GCCAATGGCAAGCTTTGT	ATGATGCCCTTTGCCTTG
L34	Cs_228_98277	A	G	TCGTCGTCGATGAGGATGT	CCAGCAGCAGCAGAAGAAG
L35	Cs_341_2998	A	G	AACCTCCACGTTCCGATG	CGACACCAGCACCAATCA
L36	Cs_165_183063	A	G	CCTGATCACGACGCACAA	GCTGAGCCTTGCCTTTGA
L37	Cs_95_59309	T	C	CTGGCAAGCGTCCTGTG	GCCCAGAGGGAAGTGTTG
L38	Cs_95_37223	A	C	GAATTGGAGCCCCATGAA	AGTGCGTTTTCGCTCCTG
L39	Cs_178_41747	A	G	TCGGTCATGATGGTCACG	CATTCTGAGCCCGACGAG
L40	Cs_177_104239	G	A	TGTGCAGCGTTCTCCTCA	GCAAAGGACTGGACCAGAA
L41	Cs_177_42727	C	T	GTCGCGATGTGGTTGTCA	CAGCAGCAGCAGCACAGT
L42	Cs_41_10227	C	T	ACCATCACCACCCAATGC	CGTCATCGCCGAAGAGAT
L43	Cs_247_29984	T	C	AAAAGCGACCCGACGACT	GCGCCAGTCCATTTCATC
L44	Cs_18_33948	C	T	CCACTGCTCTTGGGGATG	TTGCCCGTATCAGCACAG
L45	Cs_43_75161	C	T	AGCCAACCGGTTTGAATTT	ACACCCGACGAAGAGTGG
L46	Cs_52_72706	C	T	TTTGTGGGGATGCGGTAG	ATCTTGGGTGCCGTGAAG
L47	Cs_27_49147	G	A	TGCTCATGACCGTTTCCA	GCTGGGTTGAGGCTGTCT
L48	Cs_228_4669	C	A	GTCGAGCGGTCGTGATTC	GATCCCGCCGATAACACA
L49	Cs_62_56264	A	G	ATCATCGTGGGCGACATC	TTGGACAGCATGGCAGAG
L50	Cs_251_67588	T	G	ATCCAGCCCAATGCAGAG	GATTACGCGGCAAGACGTA

### QoI resistant locus genotyping and analysis

A single nucleotide polymorphism (G/C) in the Cytochrome b gene at position 143 of the *C*. *sojina* mitochondrial genome confers resistance to QoI fungicides. A custom TaqMan SNP genotyping assay was designed using the online design tools from Applied Biosystems (Thermo Scientific) and include the forward primer GGGTTATGTTTTACCTTACGGACAAATG and reverse primer GTCCTACTCATGGTATTGCACTCA and two probes to discriminate resistant and sensitive isolates: ACTGTGGCA**G**CTCATAA with VIC for the “G” resistance allele and ACTGTGGCA**C**CTCATAA with FAM for the “C” sensitive allele [21]. Each 5 μl reactions consisted of 2.5 μl 2X Taqman master mix, 0.25 μl of 20X Assay working stock (primers + probes) and 2.25 μl of genomic DNA or water as negative control. Reactions were conducted in a 384-well plate and each plate included positive controls (known resistant and sensitive) and non-template (water template) controls. Quantitative PCR (qPCR) was conducted using the QuantStudio 6 Flex Real-time PCR System (Thermo Fisher Scientific Inc.) with the following temperature settings: 95°C for 10 min for enzyme activation and 40 cycles of denaturation (95°C for 15 Sec) and annealing/extension (60°C for 1 min). Raw data was processed and scored using the QuantStudio™ Real-Time PCR Software (Thermo Fisher Scientific Inc.). Historical isolates from TN were compared with isolates from 2015 (TN) using contingency table with Fisher’s Exact test in SAS version 9.4 with the Proc GLIMMIX procedure (SAS Institute Inc., Cary, NC). Statistical analysis relating to QoI resistance from isolates collected in 2015 were conducted in JMP Pro 11.1.1 (SAS Institute Inc., Cary, NC, 1998–2012) using the LS Means Differences Student’s t-test at α = 0.05. Cultivar C3, with three isolates total, was excluded from the among cultivar analysis.

### Mating type

A previously described multiplex PCR assay was used to assign mating type (*MAT1-1-1* or *MAT1-2*) for a subset of the isolates that had unique multi-locus SNP genotypes [13]. The *MAT1-1-1* locus was amplified with CsMat1f (5’ TGAGGACATGGCCACCCAAATA) and CsMat1r (5’ AAGAGCCCTGTCAAGTGTCAGT) and the *Mat1-2* locus was amplified with CsMat2f (5’ TGTTGTAGAGCTCGTTGTTCGCA) and CsMat2r (5’ TCAGACCTTATGAGCTTGAAAGTGCT) primers [13]. The assay includes the ITS5 (5’ GGAAGTAAAAGTCGTAACAAGG) and ITS4 (5’ TCCTCCGCTTATTGATATGC) primers as an internal control to amplify the internal transcribed spacer (ITS) region [25]. The resulting PCR products were visualized under UV light on 2% agarose gel stained with GelRed (Phenix Research Products) and scored based on fragment size of *MAT1-1-1* (405 bp) and *Mat1-2* (358 bp).

### Genetic analysis

Only SNP loci with no missing data were retained for analyses. Isolates with identical multi-locus genotype were considered members of a clonal lineage and assigned a unique identifier (G1-G35) (Table 4). All subsequent analyses were conducted on the clone-corrected data except for analysis of molecular variance (AMOVA). For each marker, the allele frequencies, effective number of alleles, Nei's gene diversity, and Shannon's Information index were measured using POPGENE [26]. Population structure was assessed using Bayesian clustering in Structure 2.3.4 [27] with the following settings: no prior population information, admixture model, and allele frequency correlated. Structure was run for each possible cluster (K = 1 to 35) with 30 replications or independent runs at 1,000,000 burn-in followed by 1,000,000 Markov Chain Monte Carlo simulations. Structure Harvester was used to find the most optimal value of K (using Evanno’s method) from the results obtained from Structure [28, 29]. Distance based multivariate analysis (multiple loci and multiple genotypes) was performed to observe the relationship among different genotypes. A pairwise genetic distance matrix was computed and used for covariance-standardization and principle coordinate analysis (PCoA) in GENALEX 6.502 [30]. A phylogenetic tree of the unique genotypes was constructed using the maximum likelihood method based on the General Time Reversible model with 1000 bootstrap replications using Mega 6.06 [31, 32]. Initial tree(s) for the heuristic search were obtained by applying the Neighbor-Joining method to a matrix of pairwise distances estimated using the Maximum Composite Likelihood (MCL) approach. A discrete Gamma distribution was used to model evolutionary rate differences among sites (5 categories (+G, parameter = 4.4125)). Minimum spanning networks [33] were constructed using PopART at epsilon zero (http://popart.otago.ac.nz/). This network shows the relationship among closely related intraspecific individuals with alternative potential evolutionary paths in the form of cycles. The partitioning of genetic variance was performed with AMOVA using GENALEX 6.502. The AMOVA analysis was used to estimate molecular variance for four different groupings of the isolates including: JTN and MTN from 2015; isolates from 2015 and historical TN isolates; JTN, MTN and TN each separately; and all states separately including JTN and MTN from 2015. For Nei’s genetic distance and identity analysis, each state was treated as a group including JTN and MTN from 2015. Further examination of population differentiation was done by calculating pairwise PhiPT (which is analogous to Fst) and the Nei pairwise genetic distance and identity using GENALEX 6.502. Probability values were calculated based on 9999 permutations. The PhiPT value was also calculated for fungicide treated and untreated populations with the same settings.

**Table 4:** Summary data for 35 unique genotypes with location and number of samples, multi-locus genotypes and mating type.

Unique genotypes	Location and No. of samples	Multi-locus genotypes	Mating type
G01	GA(1)	CAAGACCCAGGCAGGCAGGACTTCACGAGTAGCAGGCCAGCTTCCCGCAG	Both
G02	JTN(1)	CAAGACTCAAGTAGGCAGGACTTTATGTACGGCAGATAAGCCTCCCGCAG	Both
G03	MTN(2)	CAAGGACTGAGTAGGGAGAATGCTGTGAGTAGCGGATAAGCTCCCTAAAG	Both
G04	AL(1)	CAATAACTAGGCAGGCGAAGCGTCATGAACAGCGGATAAGCTTCCCGCAG	Both
G05	JTN(1)	CAATGCTCAAGCAGGCAGGACTTCACGAGTAATAAACCAGCCTCCCGCAG	Both
G06	JTN(1)	CAGGAATTAAGTAGGCGAAGCGTTATGAACAGCGGATAAGCTTCCCGCAG	Both
G07	MTN(1)+TN(1)	CAGGACTCAAGCAGGCAGGACTTCACGTGTGGCAAATAAGCCTCCCGCAG	Both
G08	MTN(1)	CAGTACTCGAGCGCGGGAGGCTCCGTATGTGGCAGATAAGCTTTCCGCAG	MAT1-1-1
G09	JTN(1)+TN(1)	CGAGGACTGAACAGGCAGAATGCCGTAAGTAATGAGTAAGCTCCCCGCAG	MAT1-2
G10	MS(1)+TN(1)	CGATAACTAGACAGGCAGAACGTCATGAGTAGCAGGTAAGCTTCCCGCAG	MAT1-2
G11	IA(1)	CGATAACTGGGCAGGCAGAACGCCGTGAGTAGCGGATAAGCTTCCCGCAG	MAT1-2
G12	IL(1)	CGATACCCAGACAGGGAGGACTTCACGAGTAGCAAATAAGCCTCCCGCAT	MAT1-2
G13	AR(1)	CGATACCTAGACAGGGAGAACGTCATGAGTAGCAGGTAAGCTTCCCGCAG	Both
G14	MS(1)	CGATGACTGAACAGGCAGAATGCCGTAAGTAGCGGATAAGCTCCCTAAAG	Both
G15	JTN(2)	CGGGAATTAAGCAGGCAGAACGTCATAAGTAGCAGATAAGCTTTTCGCAG	Both
G16	MS(2)	CGGGACCCAGACAGGCAGGACTTCACGTGTGGCAAATAAGCCTCCCGCAT	MAT1-2
G17	JTN(1)	CGGTAACTGAACGGGCAGAACGCCGTGTGTGGCGGACCAGCTTCCTAAAG	Both
G18	TN(2)	TAAGACTTAAGCGGGGGAAGCTTCATGAACAGCAGATAAGCTTCCCGCAG	MAT1-1-1
G19	WI(1)	TAATGACTGGGCAGGGAGAATGTCATGAGTAATAGGTAAGCTTCCCGCAG	MAT1-1-1
G20	TN(2)	TAATGATTGAATAGGGAGAATGCTGTGAGTAGCAGATAAATTCCCCGCAG	MAT1-1-1
G21	JTN(1)	TAGGAACTAAGCAGGGAGAACGCCATGAGTAGCAAATAGGCTTCCCGCAG	MAT1-1-1
G22	JTN(77)+MTN(53)	TAGGAATCGAGCGCAGGAGGCGCCGCATGTGGCAGACCGGCTTTTCGCGG	MAT1-1-1
G23	MTN(1)	TAGGAATCGAGCGCGGGAGGCGCCGCATGTGGCAGACCGGCTTTTCGCGG	MAT1-1-1
G24	JTN(9)+MTN(1)+TN(1)	TAGGAATCGAGCGGGGGAGGCGCCGCATGTGGCAGACCGGCTTTTCGCGG	MAT1-1-1
G25	JTN(1)	TAGGGATTAAATGGGGGAAGTGTTATGAACAATAGATAGATTTCCCGCAG	MAT1-1-1
G26	LA(1)	TAGGGATTAAGTAGGGGAAGTGTTATGAACAATAGGTAGATTTCCCGCAG	Both
G27	AR(2)	TAGGGATTAAGTGGGGGAAGTGTTATGAACAATAGGTAGATTTCCCGCAG	Both
G28	JTN(1)	TAGGGCTCGAACAGGCAGGATTCCGCGTGTGGCAAATAAGCCCCCCGCAG	MAT1-1-1
G29	JTN(1)+TN(1)	TGAGGACTAAATAGGGAGAATGTTATGAGTAATGGGTAAGCTTCCCGCAG	MAT1-1-1
G30	JTN(1)	TGATAATCAGGCGCAGGAGGCGTCACATGTAGCAGACCAACTTCCCGCAG	Both
G31	TN(1)	TGATGATTAAACAGGGGAAGTGTCATAAACAGCAGATAGGCTTCCCGCAG	MAT1-1-1
G32	MTN(1)	TGATGCTCGGGCAGGGAGGATTTCACGAGTAATAAACCAGCTTCCCGCAG	MAT1-1-1
G33	JTN(1)	TGATGCTCGGGTAGGGAGGATTTTATGAGTAATAGATAAGTTTCCCGCAG	MAT1-1-1
G34	JTN(1)+MTN(1)	TGGGACTTAAGTGGGGGAAGCTTTATAAACAGCAGATAAATTTCCCGCAG	MAT1-1-1
G35	AL(1)+GA(1)	TGGGACTTAGGTAGGGGAAGCTTTATATACGGCAGATAAATTTCCCGCAG	Both

## Results

### SNP discovery and genotyping

Illumina sequencing yielded 59.4 (FLS11), 58.2 (FLS19) and 163.5 (FLS21) million paired-end reads with an average insert size of 308 bp. FLS21 was *de novo* assembled to produce draft reference contigs and the largest 144 contigs (all greater than 60kb) were used as a reference to map FLS11 and FLS19 sequences. In total, the contigs spanned 15.47 Mbp, approximately 33% of the *C*. *sojina* genome, when compared to the closely related *Cercospora zeae-maydis* (46.61 Mbp) from Joint Genome Institute. The raw reads and reference contigs are deposited in NCBI as a study accession SRP096120 and BioProject PRJNA359929, respectively. In total, 3879 candidate SNPs were identified: FLS11 (1981 SNPs) and FLS19 (1898 SNPs). Targeted-sequencing was attempted for 135 SNPs for 477 samples. A total of 186 samples were successfully genotyped for 49 SNPs and retained for further analyses. All 186 isolates with complete SNP data were genotyped for the QoI resistance locus and 41 isolates, representing all unique genotypes (see below), were tested for mating types (Table 3).

### Population structure

**Fig 1:**
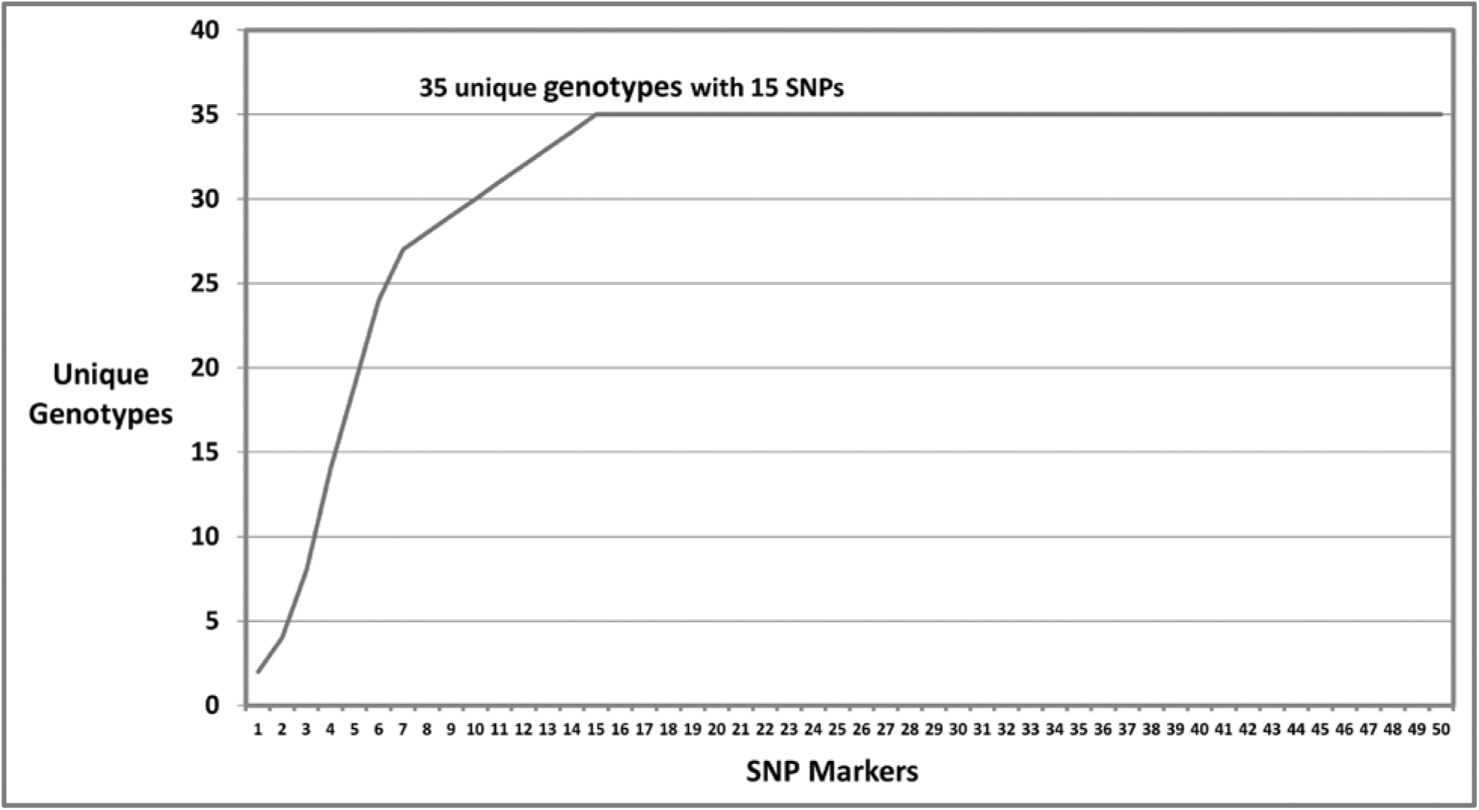
Number of unique multi-locus genotypes identified when sequentially adding SNP markers to the analysis.

Clone-correction of the 186 multi-locus genotypes revealed 35 unique genotypes (Table 4). Most of the samples (130 of 186) from Jackson and Milan in TN were clonal and belonged to genotype G22, although some isolates belonged to other genotypes (Table 4). The discriminatory power of these SNPs for identifying clonal lineages is illustrated in Fig 1. In total, 35 unique genotypes were captured by 15 SNPs. Diversity statistics for the 50 SNP markers are presented in Table 5. Each SNP locus had two alleles. The minor allele frequency (MAF) ranged from 0.057 to 0.486, with 88% of the markers having MAF > 0.1. The effective number of alleles ranged from 1.121 to 1.998, with a mean of 1.653. Gene diversity, the probability that two randomly chosen alleles from the population are different, ranged from 0.108 to 0.5, with a mean of 0.377. Shannon’s information index varied from 0.219 to 0.693, with average of 0.557.

**Fig 2:**
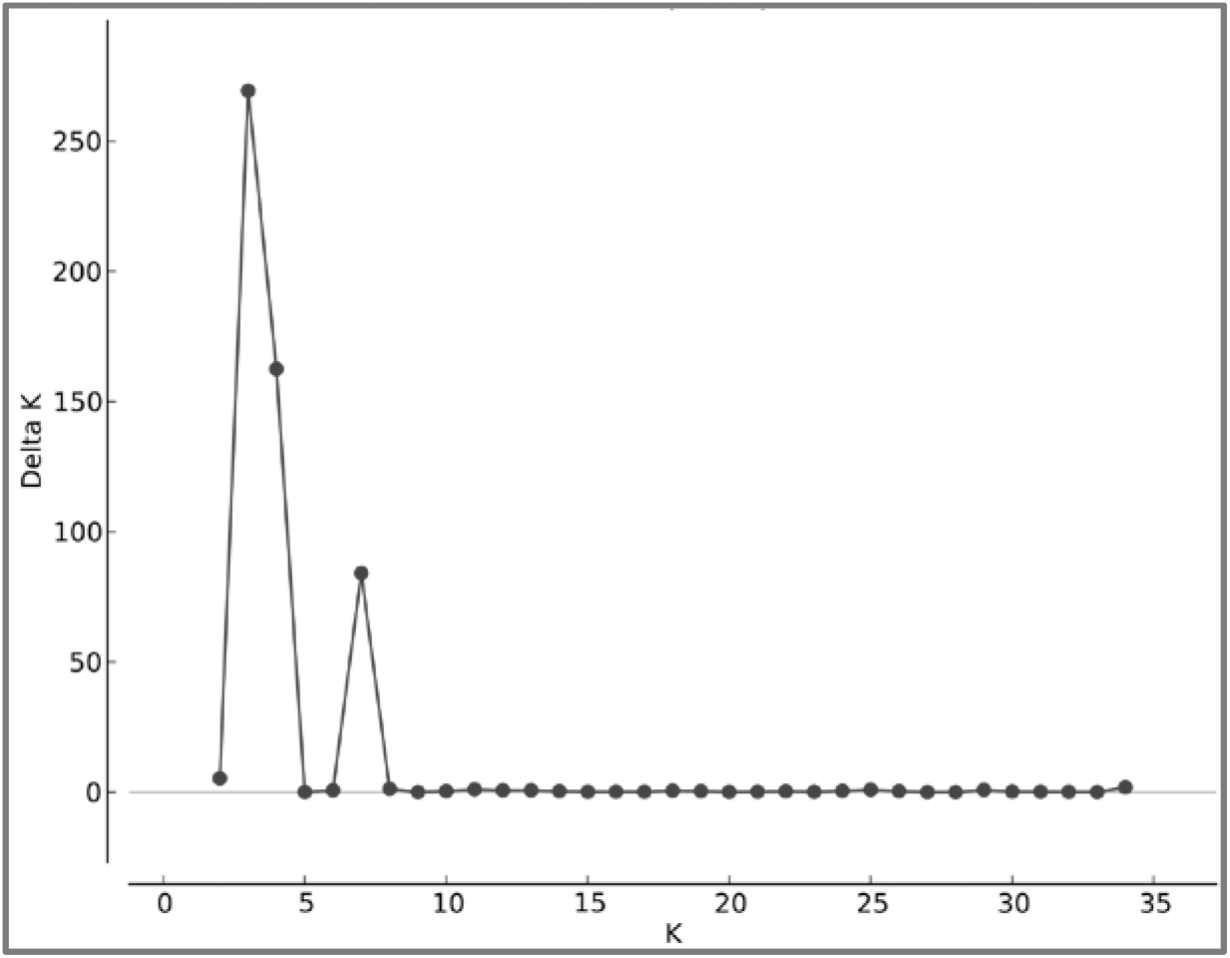
Delta K graph for each K cluster.

**Fig 3:**
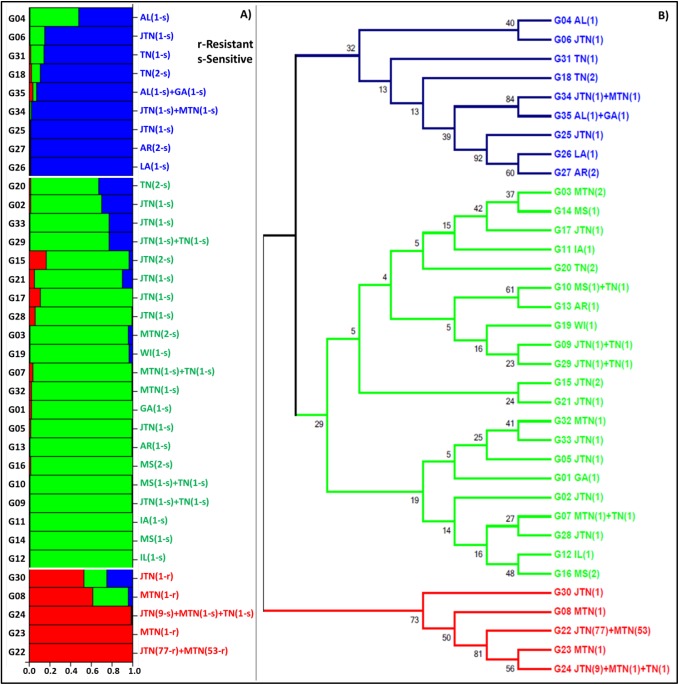
Clustering of 35 unique genotypes recovered from 186 samples of *C*. *sojina*. (A) Bayesian Structure analyses place genotypes into three clusters. Each genotype is followed by the location(s) and a number of resistant “r” or sensitive “s” isolates in brackets. (B) Maximum likelihood tree constructed using MEGA 6.06, colored to match the three groups revealed by Bayesian Structure analysis.

**Fig 4:**
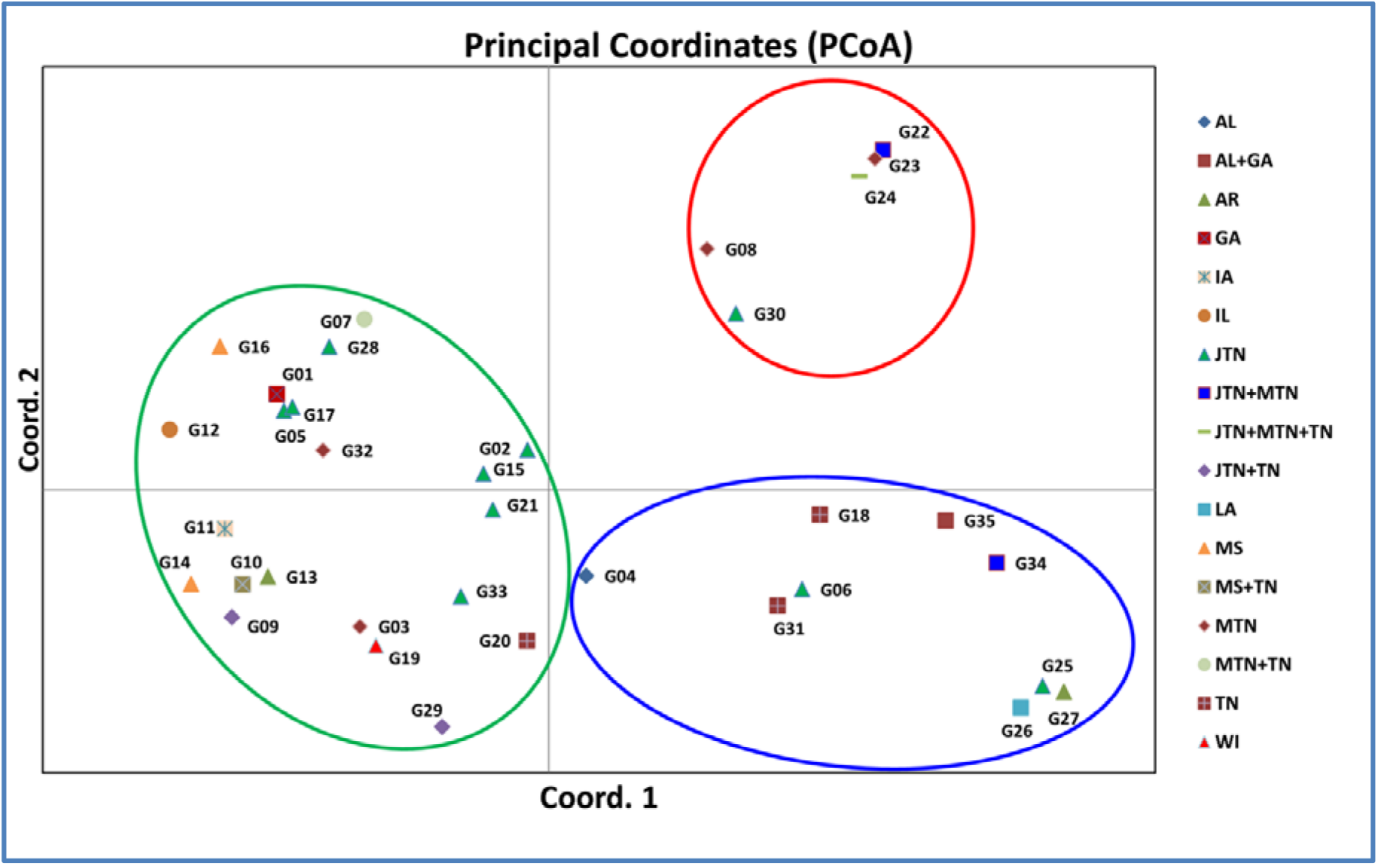
Principle coordinate analysis of 35 unique multi-locus genotypes. Key to locations is indicated on the right. Each colored circle corresponds to the colored cluster and clade in Fig 3.

Bayesian clustering of the 35 unique genotypes predicted three as the most probable K as shown in Fig 2. Grouping of the 35 unique genotypes into three clusters is shown in Fig 3A. A phylogenetic tree constructed using the maximum likelihood approach showed three distinct clades identical to the three clusters generated by Structure (Fig 3B). The three groups are also supported by the principle coordinate analysis (Fig 4).

**Table 5:** Key descriptive statistics of 50 SNPs based on 35 unique genotypes.

Locus	Markers	Allele1 frequency	Allele2 frequency	Allele1	Allele2	Na^a^	Ne^b^	h^c^	I^d^
L01	Cs_85_10076	0.514	0.486	T	C	2	1.998	0.500	0.693
L02	Cs_248_62315	0.543	0.457	A	G	2	1.985	0.496	0.690
L03	Cs_24_59400	0.543	0.457	A	G	2	1.985	0.496	0.690
L04	Cs_30_57551	0.571	0.429	G	T	2	1.960	0.490	0.683
L05	Cs_89_38914	0.600	0.400	A	G	2	1.923	0.480	0.673
L06	Cs_131_36454	0.600	0.400	A	C	2	1.923	0.480	0.673
L07	Cs_304_59625	0.600	0.400	T	C	2	1.923	0.480	0.673
L08	Cs_290_54459	0.600	0.400	T	C	2	1.923	0.480	0.673
L09	Cs_25_109428	0.600	0.400	A	G	2	1.923	0.480	0.673
L10	Cs_189_55629	0.657	0.343	A	G	2	1.820	0.451	0.643
L11	Cs_70_9315	0.657	0.343	A	G	2	1.820	0.451	0.643
L12	Cs_181_27548	0.686	0.314	C	T	2	1.758	0.431	0.623
L13	Cs_125_13742	0.714	0.286	A	G	2	1.690	0.408	0.598
L14	QoI allele	0.886	0.114	G	C	2	1.254	0.202	0.355
L15	Cs_269_60141	0.943	0.057	G	A	2	1.121	0.108	0.219
L16	Cs_386_7516	0.600	0.400	G	C	2	1.923	0.480	0.673
L17	Cs_42_10502	0.600	0.400	A	G	2	1.923	0.480	0.673
L18	Cs_42_106606	0.600	0.400	G	A	2	1.923	0.480	0.673
L19	Cs_290_2709	0.600	0.400	A	G	2	1.923	0.480	0.673
L20	Cs_419_55098	0.600	0.400	A	G	2	1.923	0.480	0.673
L21	Cs_68_11048	0.629	0.371	C	T	2	1.876	0.467	0.660
L22	Cs_131_60081	0.629	0.371	G	T	2	1.876	0.467	0.660
L23	Cs_274_24784	0.657	0.343	T	C	2	1.820	0.451	0.643
L24	Cs_181_12610	0.686	0.314	C	T	2	1.758	0.431	0.623
L25	Cs_1_66202	0.686	0.314	A	G	2	1.758	0.431	0.623
L26	Cs_128_27517	0.686	0.314	T	C	2	1.758	0.431	0.623
L27	Cs_343_52696	0.686	0.314	G	A	2	1.758	0.431	0.623
L28	Cs_119_105430	0.686	0.314	A	T	2	1.758	0.431	0.623
L29	Cs_142_22879	0.714	0.286	G	A	2	1.690	0.408	0.598
L30	Cs_142_70677	0.714	0.286	T	C	2	1.690	0.408	0.598
L31	Cs_155_10397	0.714	0.286	A	G	2	1.690	0.408	0.598
L32	Cs_157_19613	0.743	0.257	G	A	2	1.618	0.382	0.570
L33	Cs_157_39528	0.743	0.257	C	T	2	1.618	0.382	0.570
L34	Cs_228_98277	0.771	0.229	A	G	2	1.545	0.353	0.538
L35	Cs_341_2998	0.771	0.229	G	A	2	1.545	0.353	0.538
L36	Cs_165_183063	0.771	0.229	A	G	2	1.545	0.353	0.538
L37	Cs_95_59309	0.771	0.229	T	C	2	1.545	0.353	0.538
L38	Cs_95_37223	0.771	0.229	A	C	2	1.545	0.353	0.538
L39	Cs_178_41747	0.771	0.229	A	G	2	1.545	0.353	0.538
L40	Cs_177_104239	0.800	0.200	G	A	2	1.471	0.320	0.500
L41	Cs_177_42727	0.800	0.200	C	T	2	1.471	0.320	0.500
L42	Cs_41_10227	0.829	0.171	T	C	2	1.397	0.284	0.458
L43	Cs_247_29984	0.857	0.143	T	C	2	1.324	0.245	0.410
L44	Cs_18_33948	0.857	0.143	C	T	2	1.324	0.245	0.410
L45	Cs_43_75161	0.886	0.114	C	T	2	1.254	0.202	0.355
L46	Cs_52_72706	0.914	0.086	C	T	2	1.186	0.157	0.293
L47	Cs_27_49147	0.914	0.086	G	A	2	1.186	0.157	0.293
L48	Cs_228_4669	0.914	0.086	C	A	2	1.186	0.157	0.293
L49	Cs_62_56264	0.914	0.086	A	G	2	1.186	0.157	0.293
L50	Cs_251_67588	0.943	0.057	G	T	2	1.121	0.108	0.219
Mean						2	1.653	0.377	0.557

^a^ Observed number of alleles

^b^ Effective number of alleles

^c^ Nei's gene diversity

^d^ Shannon's Information index

**Fig 5:**
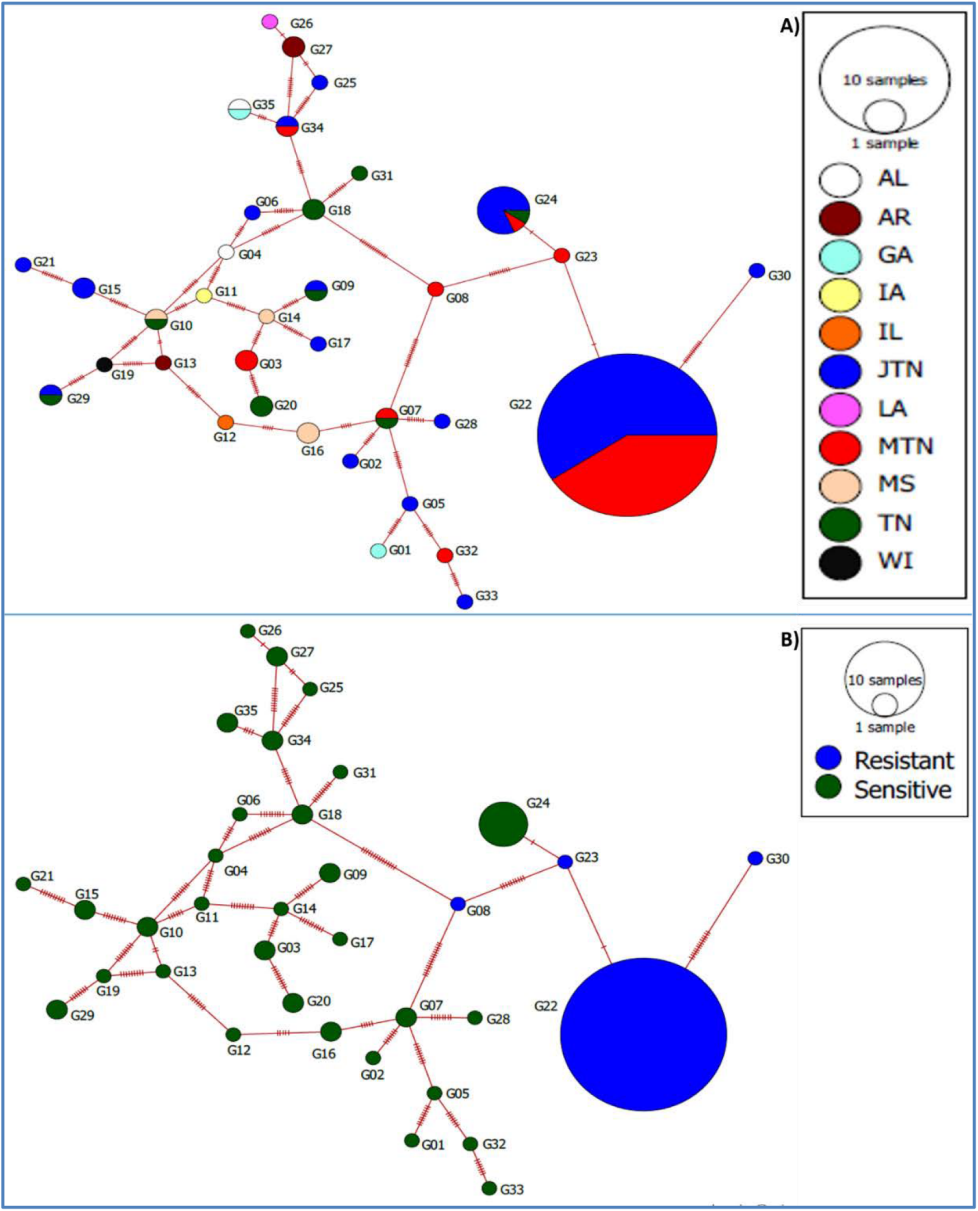
Minimum spanning networks showing the distribution of 186 isolates. (A) locations. (B) QoI sensitivity. The size of the circle represents a number of isolates with the particular genotype. Bars between circles represent the number of differences between two circles.

The Minimum spanning network provides an alternative way to visualize how the 186 samples are distributed among the 35 unique genotypes. Fig 5A is colored to indicate sample locations. Fig 5B is the same network colored to indicate the distribution of QoI resistant and sensitive isolates among the 35 unique genotypes. All 130 isolates in G22 clonal lineage were resistant with three other genotypes having resistant isolates: G08, G23, and G30. The remaining isolates fell into 31 genotypes, and all were QoI sensitive.

Genetic analysis was also performed based on locations. Locations having single samples (FL, IA, LA and WI) were not included in the analysis. The analysis was carried out among seven different locations: JTN, MTN, TN, MS, AL, AR and GA. Pairwise Nei genetic distance ranged from 0.001–0.626 with the lowest value between Jackson and Milan (Table 6). Similarly, pairwise Nei genetic identity ranged from 0.534–0.999 with maximum identity between Jackson and Milan (Table 6). AMOVA showed high genetic variance within the population and no genetic variance between JTN and MTN (Table 7). However, AMOVA showed 64.8% variance between isolates from Tennessee in 2015 and 2007, which should be assessed with caution due to the low number of isolates analyzed from 2007. Overall, the seven locations had a variance of 40.6% among populations. PhiPT analyses also indicates high population differentiation for isolates collected between 2007 and 2015 (Table 8). Fungicide treated and untreated populations had a non-significant PhiPT value (p = 0.025) and only 3% variation.

**Table 6:** Pairwise population matrix of Nei genetic distance (below the diagonal) and genetic identity (above the diagonal) among seven locations.

	JTN*	MTN	TN	MS	AL	AR	GA
**JTN**	…	0.999	0.702	0.605	0.591	0.566	0.648
**MTN**	0.001	…	0.673	0.574	0.564	0.534	0.623
**TN**	0.354	0.396	…	0.929	0.855	0.926	0.859
**MS**	0.503	0.555	0.073	…	0.824	0.880	0.869
**AL**	0.525	0.572	0.157	0.193	…	0.858	0.908
**AR**	0.568	0.626	0.077	0.127	0.153	…	0.844
**GA**	0.434	0.473	0.152	0.140	0.097	0.169	…

* Sample size: JTN(100), MTN(61), TN(10), MS(5), AL(2), AR(2), GA(2)

**Table 7:** Analysis of molecular variance (AMOVA) of *C*. *sojina* among different locations.

Source of variation	degree of freedom	Sum of Squares	Estimated Variance	Variance %
**JTN and MTN** (TN isolates from 2015)				
Among Pops	1	2.614	0.000	0.0%
Within Pops	159	464.814	2.923	100.0%
Total	160	467.429	2.923	100.0%
**JTN and MTN as a single population and TN** (TN isolates from 2015 vs 2007)				
Among Pops	1	115.805	5.978	64.8%
Within Pops	169	548.429	3.245	35.2%
Total	170	664.234	9.223	100.0%
**JTN, MTN, and TN**				
Among Pops	2	118.420	1.241	27.6%
Within Pops	168	545.814	3.249	72.4%
Total	170	664.234	4.490	100%
**JTN, MTN, TN, MS, AL, AR and GA **				
Among Pops	6	276.979	2.417	40.6%
Within Pops	175	617.614	3.529	59.4%
Total	181	894.593	5.946	100.0%

**Table 8:** Pairwise population PhiPT values (below diagonal) and P based on 9999 permutations (above diagonal).

	JTN*	MTN	TN	MS	AL	AR	GA
**JTN**	**-**	0.3283	0.0001	0.0002	0.0084	0.0077	0.0177
**MTN**	0	**-**	0.0001	0.0001	0.0039	0.0042	0.0086
**TN**	0.5995	0.6708	**-**	0.4373	0.4311	0.4862	0.4525
**MS**	0.6724	0.7479	0	**-**	0.3319	0.4327	0.3338
**AL**	0.7013	0.7800	0.02	0	**-**	0.3264	0.3344
**AR**	0.7034	0.7900	0	0	0	**-**	0.3297
**GA**	0.65	0.7500	0	0	0	0	**-**

* JTN and MTN (isolates from 2015); TN, MS, AL, AR, and GA (isolates from 2007)

### QoI resistant isolates

**Fig 6:**
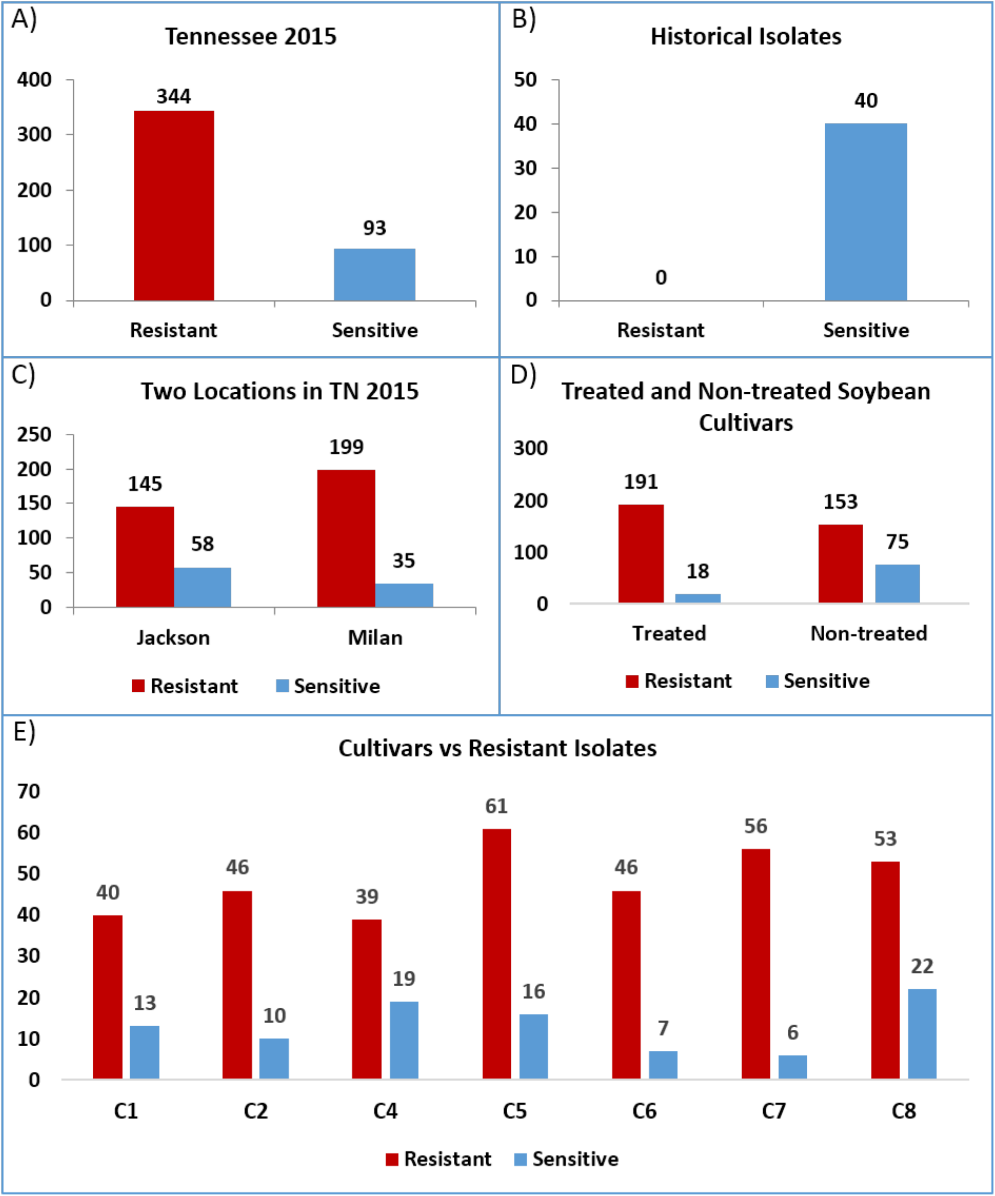
Distribution of resistant and sensitive isolates. (A) Tennessee in 2015. (B) historical collection. (C) Jackson and Milan, TN. (D) fungicide-treated and non-treated soybean cultivars. (E) Seven different soybean cultivars.

The Taqman assay successfully discriminated the resistant and sensitive alleles present in the mitochondrial Cytochrome b gene based on our positive controls. The number of QoI resistant and sensitive isolates of *C*. *sojina* recovered from treated and non-treated cultivars from Jackson and Milan are given in Table 9. Resistant isolates dominate the collection from Tennessee in 2015 (344 out of the 437 isolates tested) while all historical isolates were sensitive (Fig 6A and 6B). The Chi-square Fisher’s exact two tailed test indicates a significant increase of resistant isolates in the field in 2015 compared to isolates collected prior to 2015 (P < 0.0001). Jackson and Milan were dominated by resistant isolates, although Milan had a significantly greater proportion compared to Jackson (85% vs. 72%, respectively p<0.0001) (Fig 6C). The number of resistant isolates recovered from fungicide treated cultivars was significantly greater (91% resistant, 191 out of 209 isolates) than those collected from non-treated cultivars (67% resistant, 153 out of 228 isolatesz) (P < 0.0001) (Fig 6D). Excluding cultivar C3—VAR Beck's 393R4 (due to only 3 resistant isolates being recovered from it), the percentage of resistant isolates ranged from 68 to 90% and was not significantly different across cultivars (Fig 6E).

**Table 9:** Summary of QoI resistant and sensitive isolates recovered from fungicide treated and untreated cultivars of soybean in Jackson and Milan, 2015.

Cultivar ID	Cultivars	Jackson				Milan				Total
		Treated		Non-treated		Treated		Non-treated		
		R	S	R	S	R	S	R	S	
C1	VAR Armor 37-R33 RR2	12	5	6	5	20	1	2	2	53
C2	VAR Asgrow AG3832 GENRR2Y	7	0	7	8	20	0	12	2	56
C3	VAR Beck's 393R4	0	0	0	0	0	0	3	0	3
C4	VAR Croplan R2C 3984	14	5	6	8	11	0	8	6	58
C5	VAR Mycogen 5N393R2 RR2 g	12	0	9	11	16	1	24	4	77
C6	VAR Terral REV 39A35	7	3	11	3	13	0	15	1	53
C7	VAR USG 73P93R	22	0	5	1	12	1	17	4	62
C8	VAR Warren Seed 3780 R2Y It	14	0	13	9	11	2	15	11	75
	Total	88	13	57	45	103	5	96	30	437

### Mating-type distribution

Both mating types (*Mat1-1-1* and *Mat1-2*) were indentified (Table 10). Mating types of representative samples from the 35 unique genotypes are given in Table 4. Both mating types were identified in all three of the genetic groups described and were present in single-lesion isolates.

**Table 10:** Mating type distribution of *C*. *sojina* analyzed separately as three groups.

	Mat1-1-1	Mat1-2	Both
**Unique Genotypes**	15	5	15
**Jackson**	7	1	6
**Milan**	6	0	2

## Discussion

Our primary objective was to investigate the dynamics of FLS QoI resistance on eight cultivars, fungicide treated and untreated, at two locations in Tennessee in 2015 and to gain perspective by comparison to isolates from previous years and surrounding states. The development of novel SNP markers proved useful and it appears a subset of 15 markers was sufficient to characterize the overall genotypic diversity. Tennessee isolates from 2015 were dominated by a single QoI resistant clone, found on both fungicide treated and untreated cultivars. Although a greater proportion of resistant isolates were recovered from fungicide treated plants, there were no differences in the recovery of resistant vs. sensitive isolates among seven cultivars. Interestingly, a combination fungicide (e.g. Quadris Top SB) containing active ingredients from two different fungicide groups (Azoxystrobin from the QoI fungicide group and Difenoconazole from the DeMethylation Inhibitor (DMI) fungicide group) resulted in a higher proportion of resistant isolates, suggesting fungicide mixes can still exert selection pressure for QoI resistance. This may explain why *C*. *sojina* populations in TN have continued to increase since the first report in 2010; even when producers are using fungicides that contain active ingredients in different (non-QoI) fungicide groups (unpublished data).

Although the high levels of genetic diversity reported within fields for populations of FLS in Arkansas were not found at our two locations [13], this may be because our sampling was conducted late in the season, allowing FLS an extended period for polycyclic reproduction and dissemination. It will be useful to conduct sampling at earlier time points in the growing season to capture the full complement of genetic diversity within fields [13, 34]. The finding of both mating types in individual lesions and across all three genetic groups suggests ample opportunity for sexual recombination to play a role in shaping the population structure and to transfer the maternally inherited QoI resistance into diverse nuclear genetic backgrounds. Overall, the low level of genetic differentiation among sites was similar to the findings with *C*. *zeina*, which also had a lack of regional population differentiation [35].

Interestingly, Tennessee isolates from 2007 and 2015 shared four unique genotypes, including a second dominant genotype differing from the modern dominant clonal lineage by two SNP markers. This suggests clonal lineages may survive many years. This is a reasonable conclusion considering our data and previous studies suggesting *C*. *sojina* can remain viable in plant residues for more than two years [9, 14].

Most soybean cultivars, fungicide treated or untreated, had a high frequency of resistant isolates compared to the sensitive isolates. However, only three isolates were recovered from the FLS resistant cultivar VAR Beck's 393R4 and only from untreated plants. This suggests deployment of resistant cultivars can reduce FLS development and the further spread of QoI resistant *C*. *sojina* isolates and warrants further investigation.

Although a limited number of historical isolates were available for analyses and the population analyses are correspondingly weak, these isolates provide a useful perspective on the current situation in Tennessee. Clearly there has been a shift in the proportion of QoI resistant isolates in the last decade. Additional fine-scale sampling over time and over a wider geographical area will be useful to measure the longevity of the dominant QoI resistant clonal lineage (G22) and to investigate the role outcrossing and sexual recombination may play in the epidemiology of FLS in the face of multiple selection pressures.
